# Prostaglandin E_2_ Synthesizing Enzymes in Rheumatoid Arthritis B Cells and the Effects of B Cell Depleting Therapy on Enzyme Expression

**DOI:** 10.1371/journal.pone.0016378

**Published:** 2011-01-27

**Authors:** Karina Roxana Gheorghe, Rogier M. Thurlings, Marie Westman, Maartje J. Boumans, Vivianne Malmström, Christina Trollmo, Marina Korotkova, Per-Johan Jakobsson, Paul-Peter Tak

**Affiliations:** 1 Rheumatology Unit, Department of Medicine, Karolinska Institute, Karolinska University Hospital Solna, Stockholm, Sweden; 2 Division of Clinical Immunology and Rheumatology, Academic Medical Centre, University of Amsterdam, Amsterdam, The Netherlands; 3 Actar AB Inc., Stockholm, Sweden; University of California, San Francisco, United States of America

## Abstract

**Introduction:**

B cells may play an important role in promoting immune activation in the rheumatoid synovium and can produce prostaglandin E_2_ (PGE_2_) when activated. In its turn, PGE_2_ formed by cyclooxygenase (COX) and microsomal prostaglandin E_2_ synthase 1 (MPGES1) contributes to the rheumatoid arthritis (RA) pathological process. Therapeutic depletion of B cells results in important improvement in controlling disease activity in rheumatoid patients. Therefore we investigated the expression of PGE_2_ pathway enzymes in RA B cells and evaluated the effects of B cell depleting therapy on their expression in RA tissue.

**Methods:**

B cells expressing MPGES1 and COX-2 were identified by flow cytometry in *in vitro* stimulated and control mononuclear cells isolated from synovial fluid and peripheral blood of RA patients. Synovial biopsies were obtained from 24 RA patients before and at two consecutive time points after rituximab therapy. Expression of MPGES1, COX-1 and COX-2, as well as interleukin (IL)-1β and IL-6, known inducers of MPGES1, was quantified in immunostained biopsy sections using computerized image analysis.

**Results:**

Expression of MPGES1 or COX-2 was significantly upregulated upon stimulation of B cells from blood and synovial fluid while control cells displayed no detectable enzymes. In synovial biopsy sections, the expression of MPGES1, COX-1 or COX-2 was resistant to rituximab therapy at 8 or 16 weeks after start of treatment. Furthermore expression of IL-1β in the synovial tissue remained unchanged, while IL-6 tended to decrease after therapy.

**Conclusions:**

Therapy with B cell depleting agents, although efficient in achieving good clinical and radiographic response in RA patients, leaves important inflammatory pathways in the rheumatoid synovium essentially unaffected.

## Introduction

Rheumatoid arthritis (RA) is a chronic autoimmune disease that features persistent synovial inflammation and proliferation along with infiltration of predominantly T lymphocytes, plasma cells and macrophages. A central role for the B lymphocytes in the pathogenesis of RA is supported by the presence of autoantibodies, which are locally produced in the inflamed synovium and may promote tissue inflammation and destruction by forming immune complexes [1]. Moreover, a significant percentage of RA patients display ectopic lymphoid structures in the synovial membrane [2], [3] that could sustain T and B cell interaction [4]. Finally, effector B cells produce cytokines and other immunological mediators [5] thereby promoting the extent and direction of immune responses [6]. The observation that therapeutic B cell depletions using rituximab treatment disrupts synovial lymphoid neogenesis and decreases macrophages infiltration supports the notion that B cells orchestrate synovial inflammation in RA [7].

In the rheumatoid joint, the synovial fluid (SF) contains a variety of cytokines, chemokines, growth factors and lipid-derived mediators, which potentially mediate B cells effector functions. Of the prostaglandins, high levels are reached by prostaglandin E_2_, (PGE2) which plays a prominent role in the rheumatoid pathogenic process by promoting tissue damaging and autoimmunity [8], [9]. Microsomal prostaglandin E_2_ synthase (MPGES) 1 catalyses its formation from cyclooxygenase-derived PGH_2_ and is an inflammation-induced enzyme overexpressed in synovial tissue of RA patients [10]. MPGES1 is mostly found in fibroblast-like synoviocytes (FLS) and macrophages. Cyclooxygenase (COX) enzymes known as COX-1 and COX-2 are also widely expressed in the inflamed synovium. COX-1 is present in intimal lining layer and synovial sublining mononuclear cells and FLS [10], [11]. COX-2 has a similar localization, but is also highly expressed by endothelial cells [11]. Furthermore, whereas COX-1 expression is independent of the inflammatory status in the joint tissue, COX-2 is markedly upregulated at sites of inflammation [12]. Proinflammatory cytokines present in the rheumatic milieu, such as tumor necrosis factor (TNF), interleukin (IL) 1β [13] and IL-6 [14] are prominent inducers of MPGES1. In turn, by interacting with FLS, PGE_2_ promotes release of IL-6 [15] and matrix metalloproteinase-1 [16], thereby further sustaining a pathogenic circle.

COX-2 derived PGE_2_ also plays a central role in the humoral responses, since blocking this pathway substantially decreases antibody production [17]. PGE_2_ regulates B cell proliferation and activation [18] as well as survival [19]. This implies a possible role for PGE_2_ as a mediator of B cell immune responses in RA. To investigate this hypothesis, we studied the expression of PGE_2_-related enzymes in SF and peripheral blood (PB)-derived B cells of RA patients. Furthermore, we hypothesised that depleting B cells could change synovial immune interactions, reduce cytokine levels and decrease disease activity in the inflamed joint. These effects can in turn affect the PGE_2_ biosynthetic pathway and further contribute to decline local inflammation and clinical benefit. In this sense, it has been reported that B cells are essential in sustaining PGE_2_ production by lung macrophages [20]. Therefore, we analysed the *in vivo* effects of B cell depletion by examination of serial synovial tissue biopsies obtained before and at two consecutive points after rituximab treatment.

## Materials and Methods

### Cell preparation and flow cytometric analysis

SF and PB mononuclear cells (MC) from 10 RA patients were collected by gradient centrifugation using Ficoll-Paque (Pharmacia, Uppsala, Sweden) and stored in liquid nitrogen until assayed. Cells were cultured in RPMI medium containing 2 mM glutamine, 100 IU/mL penicillin, 100 IU/ml streptomycin and 5% human serum, at 37°C in a humidified atmosphere containing 5% CO_2_. To activate B cells, *Staphylococcus aureus* Cowan strain I and pokeweed mitogen (both from Sigma-Aldrich, Sweden) were added to the cultures at a final concentration of 10 µg/ml and 0.1 µg/ml, respectively, and cells were incubated for 16 hours. Detection of intracellular enzymes by flow cytometry was performed both in incubated cells and in unstimulated cells before culture, by using anti-MPGES1 and COX-2 antibodies according to previously described protocol [10]. Briefly, SFMC or PBMC were washed in 5% human serum, followed by staining of surface marker with anti-CD19-PerCP mouse monoclonal antibody (Becton Dickinson, San Jose, CA). Subsequently cells were fixed in 4% paraformaldehyde and incubated with rabbit polyclonal antiserum raised against MPGES1 [21] and mouse monoclonal anti-COX-2 (CX229; Cayman Chemical, Ann Harbor, MI) antibodies in saponin containing phosphate buffer, followed by addition of secondary FITC-coupled anti-rabbit or anti-mouse antibodies. B cells were identified based on scatter properties and CD19 expression and were then analyzed for expression of MPGES1 and COX-2. Due to technical issues in using COX-1 antibody for flow cytometry, we did not further pursue analysis of COX-1 in these cells.

### Patients and treatment protocol

Twenty-four patients were included from a study on the mechanism of action of rituximab in RA that was reported previously [7]. Patients had active RA (Disease Activity Score evaluated in 28 joints (DAS28≥3.2) [22] despite previous treatment with combination(s) of disease-modifying antirheumatic drugs (DMARDs) and/or TNF-blocking agents. During the study they were treated with stable dosages of methotrexate; stable treatment with non-steroidal anti-inflammatory drugs (NSAIDs) and prednisone (when taken orally in a dosage up to 10 milligrams) was allowed. The study was approved by the local Medical Ethical Committee of the Academic Medical Center/University of Amsterdam and all patients gave their written consent before participation in the study.

### Treatment and clinical evaluation

The patients were treated with two infusions of 1,000 mg rituximab (Roche, Woerden, The Netherlands) at day 1 and day 14. Methylprednisolon premedication was omitted to be able to study the specific effects of rituximab. The clinical baseline characteristics of the cohort were described previously [7]. The DAS28 was measured monthly after treatment until week 24. Response to treatment was considered according to the European League Against Rheumatism (EULAR) response criteria [23]. Responders were defined as those patients that had a good or moderate response during at least two consecutive study visits according to EULAR criteria.

### Synovial biopsy and immunohistochemistry

Synovial tissue was obtained using arthroscopy- guided synovial biopsy as described previously [24]. Biopsies were collected from the same affected joint before treatment as well as 4 weeks and 16 weeks after initiation of therapy. Immunohistochemical staining was performed on serial cryosections using MPGES1 antiserum, rabbit polyclonal anti-COX-1 (Cayman Chemical), mouse monoclonal anti-COX-2 (Cayman Chemical) antibodies, mouse anti-human CD20 (L26; DakoCytomation, Glostrup, Denmark), mouse anti-human IL-6 (B-E8; Millipore Chemicon, Billerica, MA) and mouse anti-human IL-1 (2D8; Immunokontact, Abingdon, United Kingdom). The procedure has been published earlier [25]. For surface marker staining we used the following mouse monoclonal antibodies: anti-CD22 (CLB-B-Ly; Central laboratory of The Netherlands Red Cross Blood Transfusion Centre, Amsterdam) for B cells, anti-CD3 (SK7; Becton Dickinson) for T cells, anti-CD138 (B-B4; Immunotech, Marseille, France) for plasma cells and anti-CD68 (EBM11; Dako, Glostrup, Denmark) for macrophages, as previously described [7]. Out of the 24 patients included, we have analyzed biopsy sections at all three time points in 16 patients. Due to synovial sections of insufficient quality, samples from one patient taken at week 4 and from two patients at week 16 were not included in the analysis. Additionally two patients had un-assessable tissue sections in the baseline biopsy. Three patients where we could only assess biopsy at one time point were excluded from the analysis.

Stained sections were quantitatively evaluated by computer-assisted image analysis on a Polyvar II microscope and expressed as percentage of positive staining versus total counterstained area. IL-1 and IL-6 staining was quantified as the integrated optical density, using a Leica DM-RXA light microscope [26]. Double immunofluorescence was performed on SFMC cultured on chamber slides with or without activation stimuli, using rabbit MPGES1 antiserum and a mixture of mouse monoclonal anti-CD19 and anti-CD20 antibodies (Dako Cytomation), using a protocol published earlier [21]. For immunofluorescence analysis of double stained synovial biopsy sections, samples were incubated with primary antiserum against MPGES1 and mouse monoclonal antibody against CD55 (Serotec, Oxford, UK), CD20 (Dako Cytomation) and CD138 (B-A38; Diaclone, Besancon, France), followed by addition of anti-rabbit Alexa 594 and anti-mouse Alexa 488 conjugated mouse monoclonal antibodies (both from Molecular Probes, Eugene, Oregon).

### Statistical analysis

For comparison of paired synovial biopsy samples, Wilcoxon-matched paired test for nonparametric data was used. Statistical analysis of flow cytometry data was performed using Mann-Whitney test or Wilcoxon test for paired samples and Bonferroni corrections were applied for multiple comparisons. Spearman rank correlation test for non-parametrical samples was applied for correlation analysis.

## Results

### Activated B cells from synovial fluid display a higher expression of MPGES1 and COX2 enzymes compared to peripheral blood

**Figure 1:**
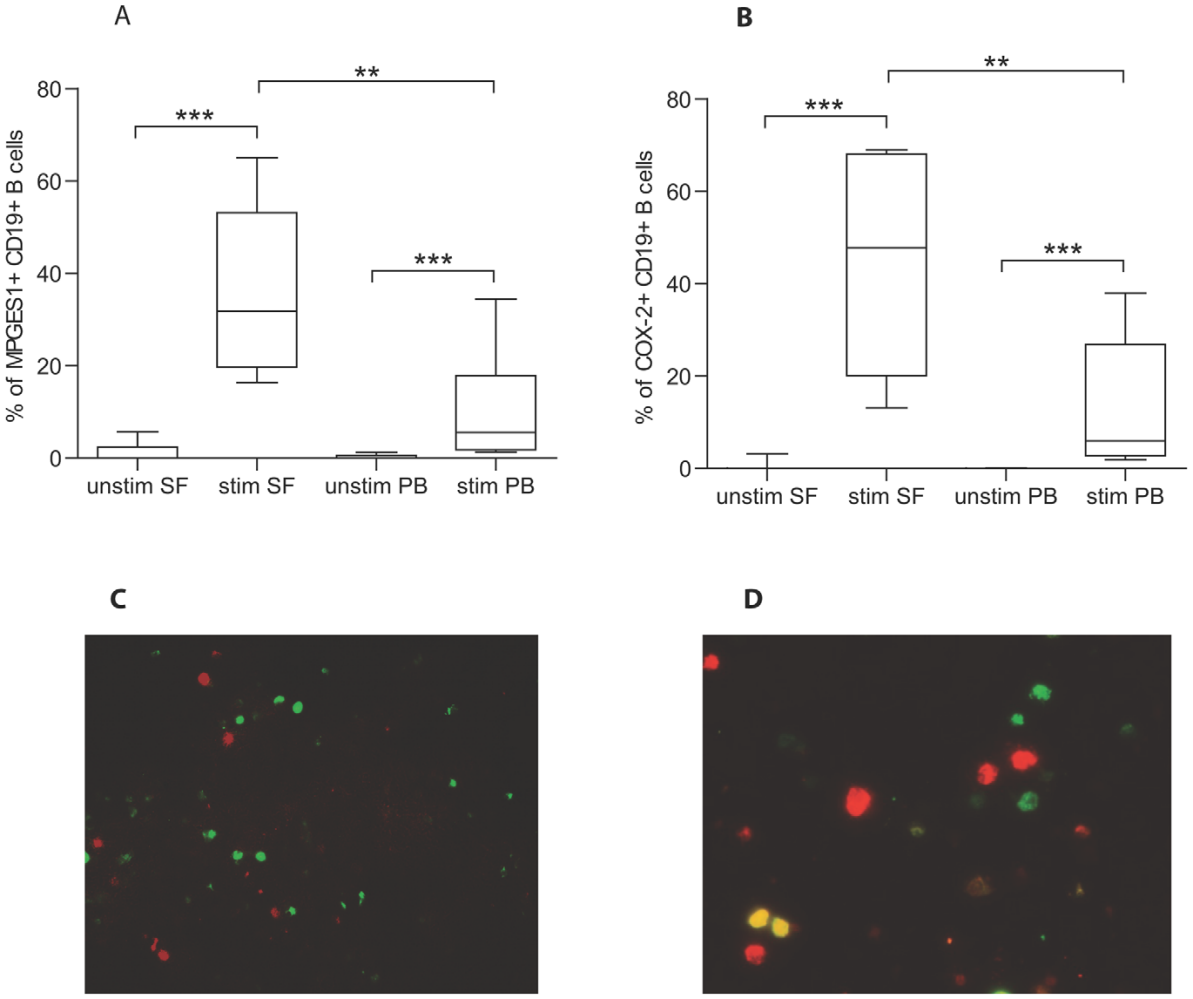
Expression of MPGES1 and COX-2 in synovial fluid (SF) and peripheral blood (PB)-derived B cells. In rheumatoid arthritis patients, SF B cells stimulated *in vitro* expressed higher levels of MPGES1 and COX-2 than PB B cells. A, Percentage of B cells that are MPGES1 positive in unstimulated and stimulated SFMC and PBMC. B, Percentage of B cells that are COX-2 positive in unstimulated and stimulated SFMC and PBMC. **  =  P<0.01, ***  =  P<0.001. C, D Immunofluorescence staining of unstimulated (C) and activated (D) RA synovial fluid cells showing CD19 positive B cells (red), MPGES1 positive cells (green) and double stained cells (yellow). Original magnification 250x.

RA B cells originating from SF or PB expressed minimal levels of MPGES1 or COX-2 after incubation without stimuli, but could significantly upregulate their expression after *in vitro* stimulation with *Staphylococcus aureus* Cowan strain I and pokeweed mitogen (Fig. 1 A, B). The expression was significantly higher in the B cell population from SF compared to PB (p = 0.002). Unstimulated B cells displayed no COX-2 expression before culture either in SF or PB (data not shown), while low MPGES1 was detected in B cells in some of the SF samples (median 0, range 0-10.2%). Confirming our flow cytometric data, we detected by immunofluorescence MPGES1 positive B cells in activated RA SFMC (Fig. 1D), but not in unstimulated cells (Fig. 1C).

### MPGES1 expression does not co-localize with B cells in rheumatoid synovium

**Figure 2:**
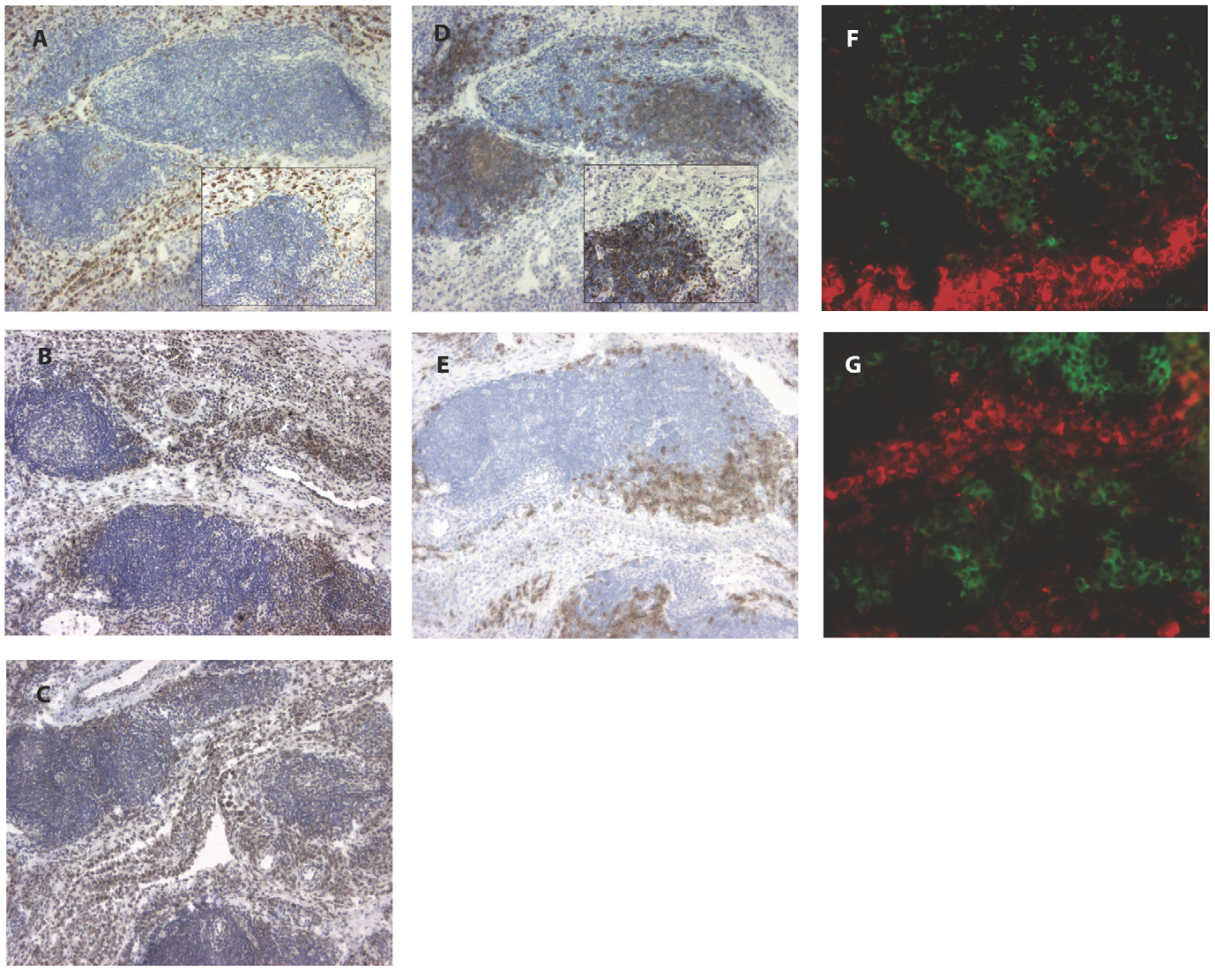
MPGES1 is not expressed in B cell-rich areas in RA synovium. Frozen sections of synovial tissue showing diaminobenzidine (brown) staining of MPGES1 (A), COX-2 (B), COX-1 (C) and CD20 B cells (D), CD138 plasma cells (E) (hematoxiline counterstained). Insets represent higher power of image A and D. Original magnification 80x and 200x (insets). (F) and (G) Immunofluorescence pictures of double stained MPGES1 (red) and CD20+ B cells (F) and CD138+ plasma cells (G) (green). Magnification 400x.

Both chromogenic and double fluorescence staining of synovial biopsies from several RA patients revealed that MPGES1 positive cells and B cells were not co-localized in the same tissue areas. As illustrated in Fig. 2, CD20 positive B cells and CD138 plasma cells have different areas of distribution compared to MPGES1 expressing cells, with virtually no overlapping. Indeed, B cells mostly accumulate inside lymphoid structures in synovial sections that display follicular synovitis, while MPGES1 positive cells can be found in intimal lining layer and synovial sublining regions, in agreement with our previous study [21]. Also, in RA biopsies not showing ectopic lymphoid neogenesis, the pattern of detected B cells is variable, from scattered isolated cells to extremely scarce (data not shown), whereas strong MPGES1 expression is virtually the rule.

Expression of COX enzymes was detected in intimal and sublining macrophages as well as in FLS (Fig. 2 B, C), as previously described [10]. In addition, cells surrounding vessels were positively stained for COX-2, while inflammatory lymphoid aggregates did not display any specific COX-2 expression. In some patients we could however detect COX-1 expressing cells within the lymphoid inflammatory infiltrates, possibly coinciding with localization of CD20 positive B cells.

### Clinical response to rituximab treatment

The DAS28 did not decrease yet at 4 weeks, but there was a statistically significant decrease in DAS28 from week 8 with a maximum mean decrease at week 20 (compared to baseline: mean (SD) decrease of 0.96 (1.1) at week 8 and 1.85 (1.4) at week 20; both p<0.001). Of the 24 patients studied, 4 patients had a good response according to EULAR response criteria and 15 patients had a moderate response, while 5 patients did not fulfil response criteria [7].

### B cell depletion therapy has limited effects on expression of MPGES1 and COX enzymes in RA synovium

**Figure 3:**
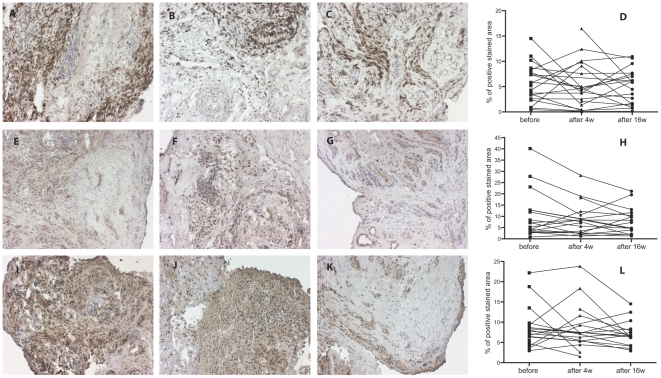
Minimal influence of rituximab treatment on expression of MPGES1, COX-1 and COX-2 in synovial tissue. Immunohistochemical staining of frozen biopsy sections from rheumatoid arthritis patients shows diaminobenzidine staining (brown) of MPGES1 (A–C), COX-2 (E-G) and COX-1 (I–K) before treatment, 4 weeks and 16 weeks after treatment (hematoxiline counterstained). Graphs depict image analysis of MPGES1 (D), COX-2 (H) and COX-1 (L) expression in stained sections from patients' biopsies at the different time points. Original magnification 150x.

Analysis of MPGES1, COX-1 and COX-2 in synovial tissue showed no statistically significant change in their expression at 4 weeks or 16 weeks after therapy initiation compared to baseline (Fig. 3).

On an individual response level, 7 patients showed an initial decrease in MPGES1 positive staining at 4 weeks, followed by a subsequent increase at 16 weeks, while 4 patients followed a different pattern with enhanced expression at 4 weeks and a decrease thereafter. In most of the patients we observed a large variability in the synovial MPGES1 response to rituximab between the different time points. A trend towards decreased COX-2 expression at 4 weeks was observed, albeit non-significant (*P* = 0.09) which was similarly maintained at 16 weeks (*P* = 0.1). COX-1 expression displayed however variable changes over time in individual patients, with no identifiable trend. Analysis of subgroups of patients not taking NSAID or GC medication showed no significant differences between groups and no consistent change in enzyme expression over time.

**Figure 4:**
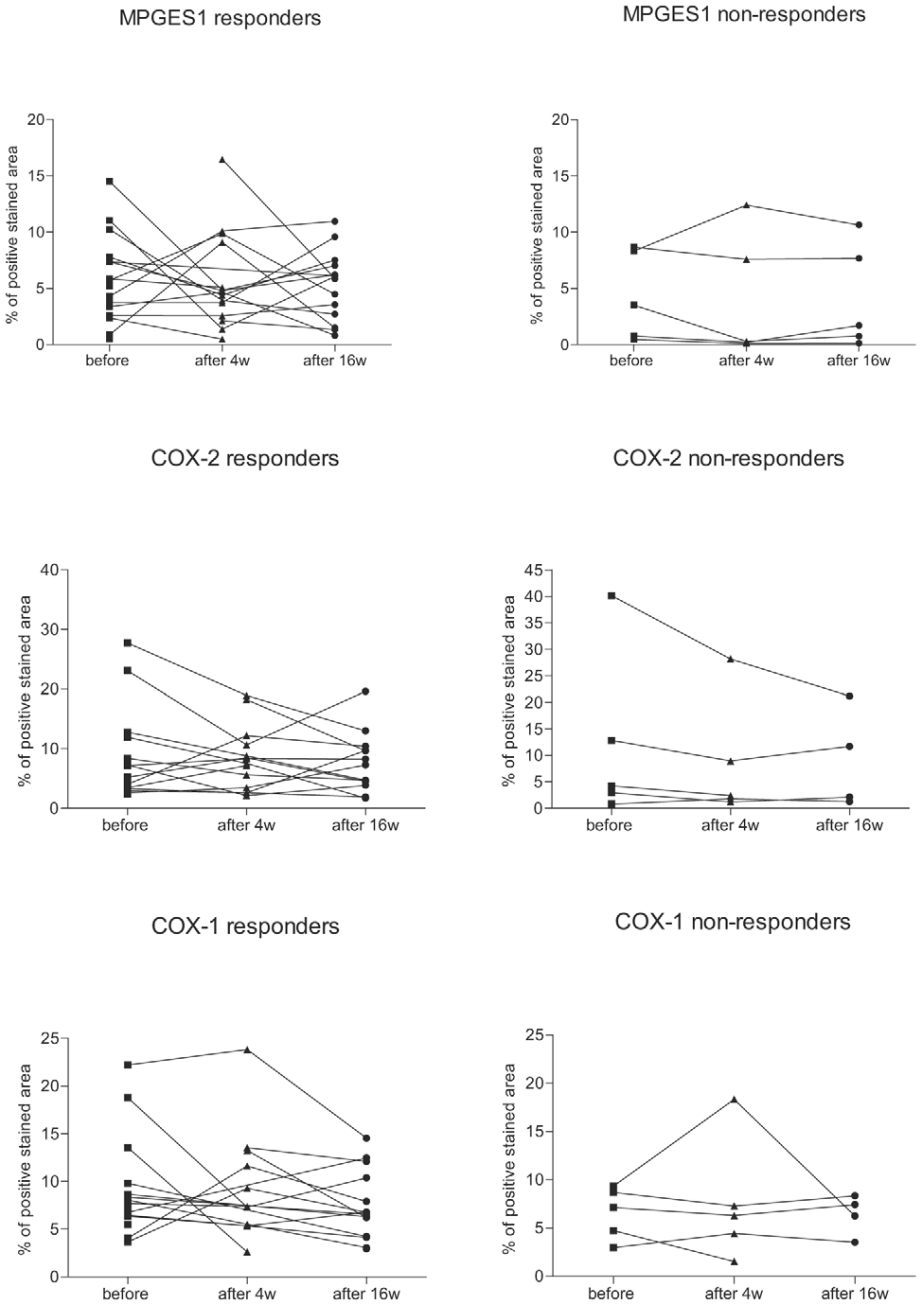
The expression of the PGE_2_ biosynthetic enzymes does not differ between responder and non-responder patients. Graphs display immunohistochemical analysis of stained biopsy sections for MPGES1, COX-1 and COX-2 expression in individual responder/non-responder patients at baseline and at 4 weeks and 16 weeks after initiation of rituximab treatment.

Next, we investigated if the synovial expression of these enzymes followed a specific pattern in responders versus non-responder patients and if the change in enzyme expression at 4 or 16 weeks is correlated with the change in inflammatory cell numbers in the tissue. As shown in Fig. 4, no specific response pattern could be observed in either of the groups, and no statistical significant difference was detected between responders and non-responders at baseline or at any of the later time points studied. Furthermore, the change in cell surface marker expression for B cells, T cells, plasma cells and lining and sublining macrophages did not correlate with the difference in enzyme expression at 4 weeks or 16 weeks following therapy start (data not shown). Thus the variability in the synovial enzyme expression was in our study not related to the clinical outcome or the inflammatory cell load in the synovial tissue.

**Figure 5:**
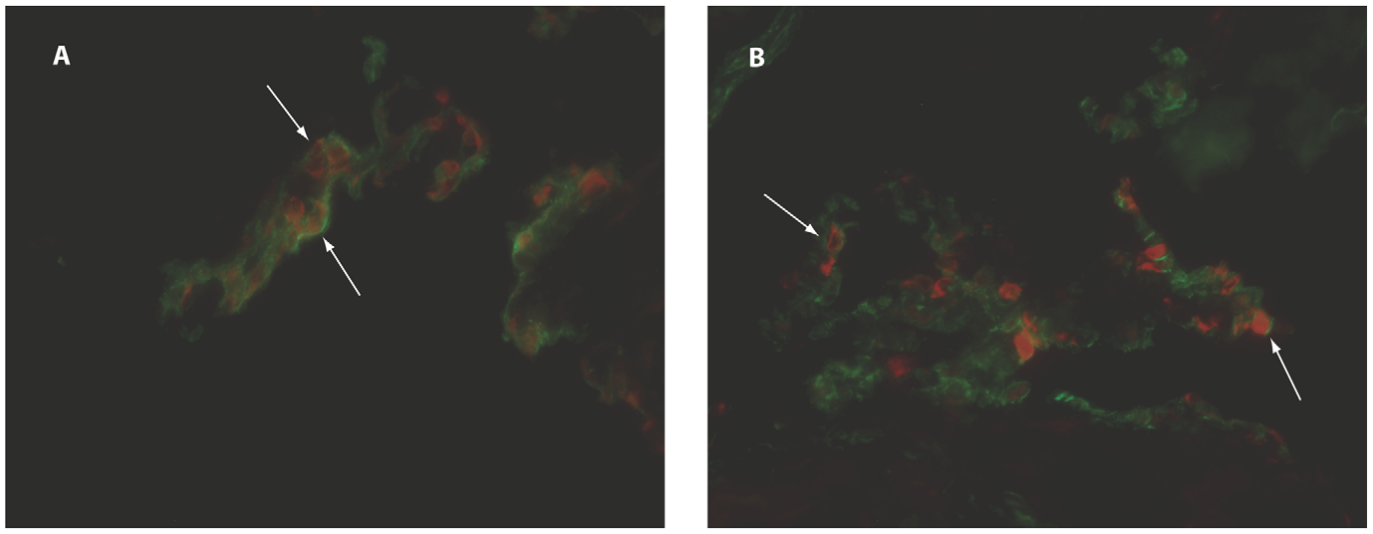
MPGES1 expression in synovial lining fibroblasts before and 16 weeks after initiation of rituximab therapy. Double immunofluorescence pictures show the presence of MPGES1 (red) expression in CD55 positive fibroblasts (green) in the rheumatoid tissue before rituximab initiation (A) and 16 weeks later (B). Original magnification 500x. Arrows point to double stained cells.

Subsequently we analyzed the cells expressing MPGES1 before and after treatment and showed that the enzyme is still present in CD55 positive FLS 16 weeks after rituximab treatment start (Fig. 5).

### B cell depletion has limited effect on MPGES1-inducing cytokines

**Figure 6:**
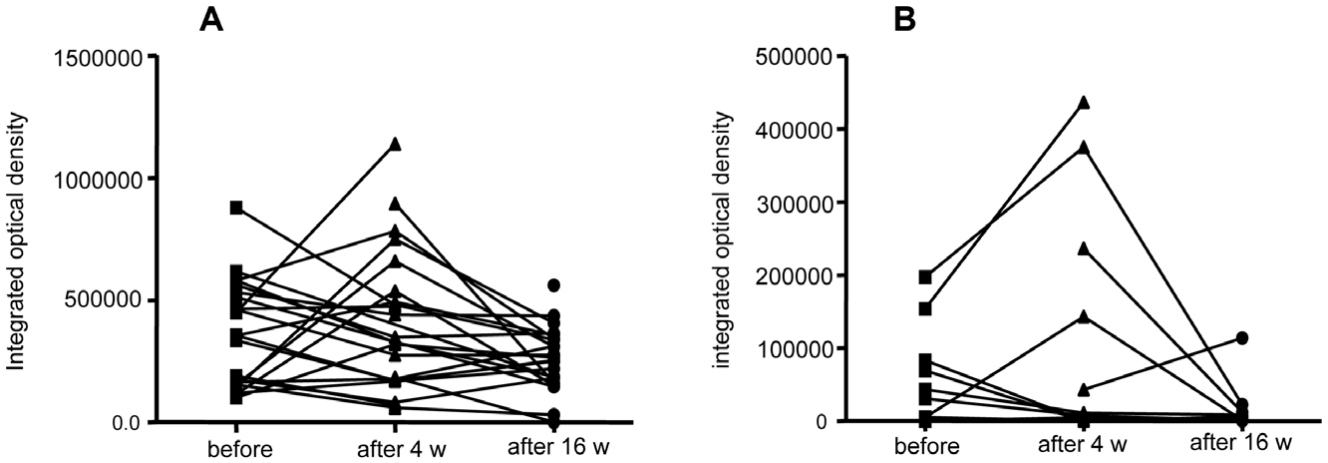
Rituximab treatment exerts limited effects on the synovial tissue expression of IL-1β and IL-6. Graphs show image analysis of positive stained sections for (A) IL-1β and (B) IL-6 before and at consecutive time points after initiation of rituximab therapy. At week 16 a trend towards decrease occurred in IL-6 but not in IL-1β.

The absent effect of rituximab on MPGES1 expression suggests a similar limited effect on the expression of MPGES1 inducing cytokines. Therefore we analyzed the change in synovial IL-1β and IL-6 expression after treatment. Before treatment IL-6 and IL-1β were present on cellular membranes and diffusely in the synovial extracellular matrix. Rituximab treatment induced a trend towards a decrease in IL-6 (*P* = 0.062) expression at week 16 but not in IL-1β (*P* = 0.12) (Fig. 6).

## Discussion

In this study we demonstrate that MPGES1 and COX-2 enzymes are upregulated in SF B cells upon activation while synovial tissue B cells do not express these enzymes. Furthermore, after B cell depletion therapy MPGES1, COX-1 and COX-2 levels in synovium of RA patients are essentially unaffected 4 weeks or 16 weeks after therapy despite clinical improvement in the majority of the studied patients. Also IL-1β and IL-6, strong inducers of MPGES1, did not change significantly.

In a previous study COX-2 was upregulated in activated PB B cells of healthy volunteers [27]. We found the same effect in SF and PB B cells from RA patients. In the synovial tissue we observed MPGES1 expression in intimal macrophages, scattered sublining macrophages and FLS. A similar distribution was observed for COX-2 with additionally positive staining around vessels. This is in line with a previous study describing MPGES1 localization in synovial tissue [21]. Indeed cells expressing MPGES1 in the synovium are mostly fibroblasts and macrophages. Also, we showed here that in RA synovial tissue MPGES1 is not present in B cell-rich areas such as the lymphoid aggregates. Despite failing to detect MPGES1 positive B cells in the rheumatoid tissue, we showed that SF and PB B cells from RA patients are able of upregulating MPGES1 and COX-2 upon *in vitro* activation while unstimulated B cells do not readily express these enzymes. These differences between *in vitro* activated SF B cells and synovial tissue B cells suggest that activation signals that are required to upregulate MPGES1 in B cells are not present in RA synovium. Moreover, we report here that activated B cells from SF have a higher expression level of MPGES1 and COX-2 than those from PB, suggesting that the local synovial compartment has a different repertoire of immune cells or a particular activation state. In fact, the proportion of B cell subsets, such as memory or naive B cells may vary between PB and SF with some phenotypes being prone to express these enzymes more than others. As such, the difference in enzyme expression that we detected may simply reflect the different abilities of the B cell subsets to respond to activation stimuli.

We showed that B cell depleting therapy exerts little effect on the PGE_2_ pathway enzymes, in agreement with our observation that B cells do not express these enzymes in the synovial tissue. However, B cells are important contributors to the inflammatory milieu in RA also by virtue of their capacity to activate T cells and secrete cytokines [28]. Since COX-2 and MPGES1 expression is inflammation-induced, a reduction of the B cell load in the rheumatoid tissue could in theory be followed by a decrease in antibody and cytokine formation and a reduced interaction with other immune cells, leading to a decrease in their activity and infiltration. Indeed a recent study of the same cohort showed that rituximab induces a decrease in the number of synovial T cells, macrophages and more heterogeneously, plasma cells [7]. In line with these changes, the inducible prostaglandin synthesis could have been affected. Of importance, our study showed that although achieving clinical improvement in a large percentage of the patients studied, rituximab did not change the local expression of MPGES1 and COX. Moreover, the variation in enzyme expression between the different time points did not reflect the change in synovial inflammatory cell populations, such as B cells, T cells, plasma cells and macrophages. In line, we found no clear cut decrease in the local expression of IL-1β and IL-6, even though these are produced by B cells, T cells and macrophages. Similarly, we have previously reported that anti-TNF agents do not suppress expression of MPGES1 or COX-2 in the rheumatoid synovium [10]. Taken together, these data indicate that important inflammatory pathways are relatively unaffected despite rituximab mediated B-cell depletion and indirect decrease in other inflammatory cells. Possibly, rituximab exerts relatively little effects on activation of the more resident cell populations in the synovium, like FLS, dendritic cells, mast cells and CD163+ macrophages. It should be noted that we cannot completely exclude the possibility of a delayed effect of rituximab on MPGES1 and COX enzymes that may become evident after 16 weeks of treatment.

Despite almost complete B cell depletion in the periphery, persistence of synovial B cells is seen in a subset of patients [29], which also correlates with infiltration with other inflammatory cells [7]. While the decrease in synovial plasma cells can predict the response to B cell depleting agents [7], persistence of plasma cell infiltration is associated with residual synovial inflammation [30]. It is hypothesized that the synovial milieu harbours molecules able to rescue B cells and promote their survival [29]. In this sense, it is worth mentioning the ability of PGE_2_ to promote survival pathways and support viability of B cells [31]. Thus the persistence of an active PGE_2_ pathway despite rituximab treatment may contribute to later relapse.

The majority of the patients included in this study received concomitant medication with NSAIDs, which can decrease the formation of PGE_2_ in synovial fluid, albeit not completely, and even affect COX-2 production, as seen in ostheoarthritis [32]. Despite representing an inherent confounding factor in such clinical studies, NSAIDs do not alter MPGES1 expression. In addition, once this medication is discontinued, COX activity may resume and account, together with MPGES1, for PGE_2_ production.

In some patients it is possible that the remaining synovial B cells may enhance PGE_2_ pathway in local fibroblasts and macrophages. Furthermore, it is noteworthy that PGE_2_ is capable of upregulating its own formation in an autocrine manner [33], [34], and thus local MPGES1 expressing cells can provide a positive feedback. Here we showed that expression of PGE_2_ related enzymes is most likely not a simple result of the local number of inflammatory cells, but of the interplay of mediators.

In conclusion, we demonstrated in this study that rituximab therapy has minimal influence on synovial expression of enzymes involved in the PGE_2_ pathway, despite clinical response in most RA patients. Furthermore, recent reports have demonstrated that optimal antibody production by B cells needs COX-2-derived PGE_2_ [17], implying that blocking this pathway may possibly lead to reduced antibody formation in synovial tissue and thus less joint damage. Together, these data may provide one explanation for the fact that rituximab treatment, similar to TNF blockade, does not induce complete remission in the majority of the RA patients and suggest that blocking the PGE_2_ pathway by targeting MPGES1 may lead to novel therapeutical strategies and complement current anti-rheumatic therapy by providing additional benefit in controlling the inflammatory process in the rheumatoid joint.
